# The Development and Validation of the Bergen–Yale Sex Addiction Scale With a Large National Sample

**DOI:** 10.3389/fpsyg.2018.00144

**Published:** 2018-03-08

**Authors:** Cecilie S. Andreassen, Ståle Pallesen, Mark D. Griffiths, Torbjørn Torsheim, Rajita Sinha

**Affiliations:** ^1^Faculty of Psychology, University of Bergen, Bergen, Norway; ^2^Psychology Department, Nottingham Trent University, Nottingham, United Kingdom; ^3^Yale Stress Center, Yale University School of Medicine, New Haven, CT, United States

**Keywords:** hypersexuality, sexual addiction, measurement development, psychometric scale, five-factor model of personality, narcissism, self-esteem, demographics

## Abstract

The view that problematic excessive sexual behavior (“sex addiction”) is a form of behavioral addiction has gained more credence in recent years, but there is still considerable controversy regarding operationalization of the concept. Furthermore, most previous studies have relied on small clinical samples. The present study presents a new method for assessing sex addiction—the Bergen–Yale Sex Addiction Scale (BYSAS)—based on established addiction components (i.e., salience/craving, mood modification, tolerance, withdrawal, conflict/problems, and relapse/loss of control). Using a cross-sectional survey, the BYSAS was administered to a broad national sample of 23,533 Norwegian adults [aged 16–88 years; mean (± *SD*) age = 35.8 ± 13.3 years], together with validated measures of the Big Five personality traits, narcissism, self-esteem, and a measure of sexual addictive behavior. Both an exploratory and a confirmatory factor analysis (RMSEA = 0.046, CFI = 0.998, TLI = 0.996) supported a one-factor solution, although a local dependence between two items (Items 1 and 2) was detected. Furthermore, the scale had good internal consistency (Cronbach's α = 0.83). The BYSAS correlated significantly with the reference scale (*r* = 0.52), and demonstrated similar patterns of convergent and discriminant validity. The BYSAS was positively related to extroversion, neuroticism, intellect/imagination, and narcissism, and negatively related to conscientiousness, agreeableness, and self-esteem. High scores on the BYSAS were more prevalent among those who were men, single, of younger age, and with higher education. The BYSAS is a brief, and psychometrically reliable and valid measure for assessing sex addiction. However, further validation of the BYSAS is needed in other countries and contexts.

## Introduction

In recent years research into frequent and persistent problematic sexual behavior has increased (Kraus et al., 2016). This out-of-control, excessive, and problematic sexual behavior has been described using many different labels including (amongst others) hypersexuality, sexual compulsivity, sexual impulsivity, erotomania, nymphomania (in women), satyriasis (in men), sexual addiction, and sexual dependency (Kafka, 2010; Karila et al., 2014; Kingston, 2015; Wéry and Billieux, 2017). There has been much debate over many years as to whether this behavior is best conceptualized as an obsessive-compulsive disorder, an addiction, or a disorder of impulse-control (Karila et al., 2014; Piquet-Pessôa et al., 2014), and consequently been explained according to different conceptual models (Campbell and Stein, 2015; Kingston, 2015).

In the wake of new research suggesting that sex has an addictive potential—most probably mediated by brain circuits and neurotransmitters that are known to be involved in the experience of reward and euphoria—the conceptual interest in hypersexuality as an addiction has rapidly grown (Holstege et al., 2003; Hamann et al., 2004; Goodman, 2008; Griffiths, 2012; Kor et al., 2013; Karila et al., 2014; Voon et al., 2014; Kingston, 2015). In this context, “*sex addiction”* can be defined as being intensely involved with sexual activities (e.g., fantasies, masturbation, intercourse, pornography) across different media (cybersex, telephone sex, etc.). Furthermore, those with the condition report their sexual motivation is uncontrollable, and that they expend a lot of time both thinking about and being engaged in sexual activities that negatively affects many other areas in their lives.

“Sex addiction” is currently not listed in the psychiatric taxonomy. However, the *International Classification of Disease* (*ICD-10*; World Health Organization, 1992), included excessive sexual drive and excessive masturbation as diagnoses, divided into satyriasis (for men) and nymphomania (for women), whereas “compulsive sexuality” is currently being considered (as an impulse-control disorder) for inclusion in the upcoming *ICD-11* (Grant et al., 2014). The latest (fifth) edition of the *Diagnostic and Statistical Manual of Mental Disorders* (*DSM-5*; American Psychiatric Association, 2013) has increased its recognition of non-chemical addictions (Petry, 2015) with the inclusion of Gambling Disorder as a behavioral addiction within the main text and Internet Gaming Disorder in the section Results appendix (condition for further study). Although sex addiction (in the form of “hypersexual disorder”) was proposed (Kafka, 2010) and evaluated by the *DSM-5* task force, along with a set of empirically tested criteria (Kafka, 2010; Reid et al., 2012), it was rejected due to lack of research into diagnostic criteria and a split view on how to conceptualize the disorder (Kafka, 2013; Campbell and Stein, 2015).

In line with this, a limitation of prior research is the absence of a general consensus about how sex addiction should be determined, understood, and assessed (Reid, 2016). Thus, unreliable prevalence estimates among non-representative (self-selected convenience) samples spanning from 3 to 17% (and higher) have been reported. In terms of demographic variables, research has shown a relatively consistent positive relationship between sex addiction and young age, male gender, single status, and high education (for recent reviews see Kafka, 2010; Sussman et al., 2011; Karila et al., 2014; Campbell and Stein, 2015; Wéry and Billieux, 2017). However, it has been argued that women have been largely underrepresented in this field of research, and consequently little is known about their pattern of sex addiction (Dhuffar and Griffiths, 2014, 2015; Klein et al., 2014).

Research has associated sex addiction with personality factors representative of other addictive behaviors (Karila et al., 2014), including high levels of extroversion and neuroticism and low levels of conscientiousness and agreeableness (Schmitt, 2004; Pinto et al., 2013; Rettenberger et al., 2016; Walton et al., 2017). These characteristics refer to personalities who are highly sensation seeking, emotionally reactive, spontaneous, and inconsiderate, as opposed to being low-keyed, emotionally stable, self-disciplined, and concerned for social harmony. The limited research employing the five-factor model of personality (Costa and McCrae, 1992; Wiggins, 1996) in this context has found the trait openness to experience to be unrelated to sex addiction (Schmitt, 2004; Pinto et al., 2013; Rettenberger et al., 2016; Walton et al., 2017). However, it seems more likely that “liberal personalities” who appreciate “borderline” experiences are more at risk for sex addiction, than traditional, close-minded and cautious people (e.g., Elmquist et al., 2016). Addictive sex behaviors have also frequently been positively related to narcissism (Black et al., 1997; Raymond et al., 2003; Kafka, 2010; Kasper et al., 2015) and negatively related to self-esteem (Cooper et al., 1999, 2004; Delmonico and Griffin, 2008; Kor et al., 2014; Doornwaard et al., 2016).

The growing interest in “sex addiction” both conceptually and empirically has been accompanied with a rapid development of instruments such as the Sexual Addiction Screening Test (SAST; Carnes, 1989) and SAST–Revised (SAST–R; Carnes et al., 2010), the Shorter PROMIS Questionnaire–sex subscale (SPQ-S; Christo et al., 2003), PATHOS^1^ (Carnes et al., 2012), and the Short Internet Addiction Test (Young, 1998) adapted to online sexual activities (s-IAT-sex; Laier et al., 2013; Pawlikowski et al., 2013; Wéry et al., 2016a). While other validated scales have been developed, they assess and conceptualize “hypersexuality” as a compulsive, impulsive, and/or sexual dysregulation disorder (e.g., Kalichman and Rompa, 1995; Coleman et al., 2001; Reid et al., 2011).

The aforementioned scales vary greatly in terms of development procedure, item structure, cut-off score, and psychometric properties (Hook et al., 2010; Karila et al., 2014; Campbell and Stein, 2015; Wéry and Billieux, 2017), and have primarily been investigated in small non-representative clinical and targeted samples (Karila et al., 2014). Some are highly population-specific (e.g., male, female, gay; Carnes, 1991; O'Hara and Carnes, 2000; Carnes and Weiss, 2002), whereas others are highly content-specific (e.g., online sexual behavior; Carnes et al., 2010; Wéry et al., 2016a). Widely used scales (e.g., SAST-R, PATHOS) also include items that are arguably inappropriate with regards to defining sex addiction [i.e., “*Were you sexually abused as a child or adolescent?*,” “*Did your parents have trouble with sexual behavior?*” (SAST; Carnes, 1989, pp. 218–219), “*Have you ever sought help for sexual behavior you did not like?*” (PATHOS; Carnes et al., 2012, p. 11)]. The SAST-R (Carnes et al., 2010) and PATHOS (Carnes et al., 2012) employ a dichotomous yes/no response format, whereas empirical research suggests that the dimensional/continuum assessment of problematic sexual behavior should be part of clinical diagnostic practice (Winters et al., 2010; Walters et al., 2011; Carvalho et al., 2015). Current scales that assess problematic sexual behavior tend to be relatively lengthy. More specifically, Womack et al. (2013) reported a mean of 32.5 items (*SD* = 34.2) when systematically reviewing 24 self-report hypersexuality measures. However, applicable measures should satisfy key criteria (such as brevity; Koronczai et al., 2011), particularly among impulsive populations who are more likely to value and participate in activities that are short-lasting.

An arguably major limitation of current scales is that the items assessing addictive sexual behavior do not reflect central addiction components (Brown, 1993; Griffiths, 2005). Such criteria have been used as a framework for developing a number of psychometric scales for various behavioral addictions including work addiction (Andreassen et al., 2012a), gaming addiction (Lemmens et al., 2009), shopping addiction (Andreassen et al., 2015), exercise addiction (Terry et al., 2004), and social media addiction (Andreassen et al., 2016). In relation to sex addiction, these symptoms would be: *salience/craving*—over-preoccupation with sex or wanting sex, *mood modification*—excessive sex causing changes in mood, *tolerance*—increasing amounts of sex over time, *withdrawal—*unpleasant emotional/physical symptoms when not having sex, *conflict*—inter-/intrapersonal problems as a direct result of excessive sex, *relapse*—returning to previous patterns after periods with abstinence/control, and *problems*—impaired health and well-being arising from addictive sexual behavior.

Current scales commonly capture some of the aforementioned symptoms, but do not cover them all (e.g., PATHOS and SAST-R). One reason for this may be that previously developed scales were inspired by three prominent sets of proposed criteria identified in the literature. These are (i) Carnes' 1991 criteria that exclude withdrawal and salience, (ii) Goodman's (1998) criteria that exclude mood modification, and (iii) Kafka's (2010, 2013) criteria that do not include tolerance, mood modification, salience, and withdrawal (Wéry and Billieux, 2017). The s-IAT-sex scale (Laier et al., 2013; Pawlikowski et al., 2013; Wéry et al., 2016a) includes all core addiction criteria, but was specifically developed to assess online sex addiction only. While modern Internet applications may facilitate and enhance the emergence of addictive sex behavior due to factors such as convenience, anonymity, accessibility, and disinhibition (Griffiths, 2012; Wéry and Billieux, 2017), there is arguably a demand for a brief and psychometrically sound assessment measure that determines sex addiction irrespective of place, context, and population.

Given the aforementioned findings and debates in the field, the present study explored the psychometric properties of a new brief sex addiction measure, the Bergen–Yale Sex Addiction Scale (BYSAS), consisting of items constructed on the basis of core criteria that have been emphasized across several behavioral addictions and that uses established addiction frameworks to highlight the content validity (Brown, 1993; Griffiths, 2005; American Psychiatric Association, 2013; Andreassen et al., 2013). It was expected that the new instrument would be highly correlated with similar constructs (i.e., convergent validity) and correlate poorly with dissimilar constructs (i.e., discriminant validity; Nunnally and Bernstein, 1994). Six hypotheses were examined. These were that:

*Hypothesis 1*. The BYSAS has a one-factor structure with high factor loading (> 0.60) for all scale items, and all indexes (root mean square error of approximation [RMSEA] < 0.06, comparative fit index [CFI] and Tucker-Lewis index [TLI] > 0.95; Hu and Bentler, 1999) showing good data fit.
*Hypothesis 2*. The BYSAS has a high internal consistency (Cronbach's alpha > 0.80).
*Hypothesis 3*. The BYSAS correlates positively with another measure of addictive sex behavior (SPQ-S; Christo et al., 2003).
*Hypothesis 4*. The BYSAS score is positively related to being male, single and higher educated, and inversely related to age.
*Hypothesis 5*. The BYSAS score is positively related to neuroticism, extroversion, and openness, and negatively related to agreeableness and conscientiousness.
*Hypothesis 6*. The BYSAS score is positively related to narcissism and negatively related to self-esteem.

## Materials and methods

### Procedure

Data were collected through a web-based cross-sectional survey assessing excessive behaviors. The survey was broadcasted in the online edition of five different nationwide Norwegian newspapers during spring 2014. In order to participate, respondents were instructed to click on an online link. All respondents had to be at least 16 years of age. Information about the study was provided on the webpage. The respondents were informed that they would receive an automatically generated feedback based on their scores as well as an interpretation related to several of the scales upon completion of the survey. No material/monetary incentive was provided. All data were stored on a server hosted by a company administering such surveys for the researchers (www.surveyxact.no). One week following study initiation, all collected data were forwarded to the research team.

In total, 23,533 individuals completed all items of the survey (and were retained for analysis). Participation was voluntary, anonymous, confidential, and non-interventional, and followed the ethical guidelines of the Helsinki Declaration and the Norwegian Health Research Act. The Institutional Review Board of the Faculty of Psychology, University of Bergen, approved the study.

### Participants

The mean age of participants (*N* = 23,533) was 35.8 years (*SD* = 13.3), ranging from 16 to 88 years. In terms of included age groups, the majority of the participants were aged 16–30 years (40.7%) followed by those aged 31–45 years (35%), 46–60 years (19.8%), and over 60 years (4.5%). The sample comprised 15,299 women (65%) and 8,234 men (35%). In terms of relationship status, 15,373 (65.3%) were currently in a relationship (i.e., married, common law partner, partner, boyfriend, or girlfriend) and 8,160 (34.7%) were not (i.e., single, divorced, separated, widow, or widower). With regard to education, 2,350 had completed compulsory school (10%), 5,949 had completed high school (25.3%), 3,989 had completed vocational school (17%), 7,630 had a Bachelor's degree (32.4%), 3,343 had a Master's degree (14.2%), and 272 had a PhD degree (1.2%).

### Measures

#### Demographics

Participants completed one-item measures of demographics (i.e., age, gender, relationship status, highest completed education) by using a closed-ended response format.

#### Bergen–yale sex addiction scale (BYSAS)

The BYSAS was developed utilizing the six addiction criteria emphasized by Brown (1993), Griffiths (2005), and American Psychiatric Association (2013) encompassing salience, mood modification, tolerance, withdrawal symptoms, conflicts and relapse/loss of control. One item was created for each single criterion. More specifically, the criteria included items relating to salience/craving (i.e., preoccupation with sex/masturbation), mood modification (i.e., sex/masturbation improves mood), tolerance (i.e., more sex/masturbation is required in order to be satisfied), withdrawal symptoms (i.e., reduction or preclusion from sex/masturbation create restlessness and negative feelings), conflict/problems (i.e., sex/masturbation creates conflicts and cause some kind of problem), and relapse/loss of control (i.e., return to old sex/masturbation patterns after a period of control or absence). The specific wording of the items and the response alternatives were based on the wording and response alternatives used in scales assessing other behavioral addictions (Andreassen et al., 2012b). The time frame concerned the past year using a 5-point Likert response format (0 = *very rarely*, 1 = *rarely*, 2 = *sometimes*, 3 = *often*, and 4 = *very often;* see **Appendix A** for complete list of items and response formats for the BYSAS), yielding a composite BYSAS score ranging from 0 to 24 (see Table 1). In order to be operationally classed as a “sex addict” in the present study, the symptoms had to be present at a specific level/magnitude [defined as scoring at least 3 (*often*) or 4 (*very often*)]. This is in line with the way cut-offs have been operationalized for other scales assessing behavioral addictions (e.g., Lemmens et al., 2009; Andreassen et al., 2012b). In addition, a specific number of criteria (often more than half) had to be endorsed (here “often” or “very often”) to be classed as an addiction (American Psychiatric Association, 2013). In this case at least four of the six BYSAS items had be endorsed in order to regard the participant as a sex addict. Scoring 0 on the composite BYSAS-score was defined as “no sex addiction” which seems reasonable as these participants answer “never” to all the six items. A composite score between 1 and 6 was defined as “low sex addiction risk” as these participants maximally could score above cut-off on two of the six items. Those with a composite score of 7 or above but did not fulfill the criteria for sex addiction were defined as having “moderate sex addiction risk”. This label seems suitable as this equals a mean score above 1 on all six items.

**Table 1:** The distribution of scores, mean score and standard deviation (*SD*) on the six items of the Bergen-Yale Sex Addiction Scale (BYSAS) for males (♂, *n* = 8,234), females (♀, *n* = 15,299), and the whole (=) sample (*N* = 23,533).

**Items**			**Frequency (%)**					**Mean**	***SD***
***How often during the past year have you…***			**0**	**1**	**2**	**3**	**4**		
1.	Spent a lot of time thinking about sex/masturbation or planned sex? [BYSAS1 on salience—craving]	♂ ♀ =	20.5 52.6 41.4	19.0 20.1 19.7	31.7 19.4 23.7	20.0 6.1 11.0	8.7 1.7 4.2	1.78 0.84 1.17	1.23 1.05 1.20
2.	Felt an urge to masturbate/have sex more and more? [BYSAS2 on tolerance]	♂ ♀ =	26.4 58.7 47.4	24.3 19.9 21.4	28.4 15.4 20.0	14.8 4.7 8.3	6.1 1.3 3.0	1.50 0.70 0.98	1.20 0.98 1.13
3.	Used sex/masturbation in order to forget about/escape from personal problems? [BYSAS3 on mood modification]	♂ ♀ =	59.3 76.6 70.6	17.5 11.8 13.8	14.4 8.4 10.5	5.7 2.4 3.5	3.1 0.8 1.6	0.76 0.39 0.52	1.09 0.80 0.93
4.	Tried to cut down on sex/masturbation without success? [BYSAS4 on relapse—loss of control]	♂ ♀ =	67.0 92.2 83.4	16.3 5.3 9.2	10.6 1.6 4.7	4.2 0.6 1.8	1.9 0.3 0.9	0.58 0.11 0.28	0.97 0.45 0.71
5.	Become restless or troubled if you have been prohibited from sex/masturbation? [BYSAS5 on withdrawal symptoms]	♂ ♀ =	53.0 81.5 71.5	21.0 10.1 13.9	16.4 6.0 9.6	6.8 1.8 3.5	2.8 0.6 1.4	0.85 0.29 0.49	1.10 0.71 0.91
6.	Had so much sex that it has had a negative impact on your private relationships, economy, health or job, studies? [BYSAS6 on conflict—problems]	♂ ♀ =	87.1 96.3 93.0	7.8 2.5 4.4	3.3 0.8 1.7	1.0 0.3 0.5	0.9 0.1 0.4	0.21 0.05 0.11	0.63 0.31 0.46

*Scale ranged from 0—“very rarely” to 4—“very often.” The mean composite score for the whole sample was 3.54 (SD = 4.14). Composite score range 0–24*.

#### Shorter PROMIS questionnaire—sex subscale

The Shorter PROMIS Questionnaire [SPQ; Christo et al., 2003 (PROMIS Questionnaire; Lefever, 1988)] is a psychometrically validated measure of 16 (chemical and non-chemical) addictive behaviors, including sex (e.g., Haylett et al., 2004; Pallanti et al., 2006; MacLaren and Best, 2010, 2013). Participants completed the sex subscale of the SPQ using a 6-point scale [0 = *not like me at all* and 5 = *most like me*; 10 items: *M* = 13.44, *SD* = 7.14, α = 0.90; sample item: “*I would take an opportunity to have sex despite having just had it with somebody else*” (see **Appendix B** for the full list of items)]. The sex subscale of the SPQ (hereafter referred to as the SPQ-S) assesses some aspects of reward seeking and compulsion, including some potentially addictive behaviors and symptoms of sex disorder. However, it only assesses addictive tendencies toward sexual intercourse/activities (with others), and also excludes core addiction criteria. The 10 items of the SPQ-S were translated from English to Norwegian separately by the Norwegian authors of the present study.

#### Big five

The Mini-International Personality Item Pool (Mini-IPIP; Donnellan et al., 2006) was used to assess personality, and is a psychometrically acceptable and practically useful short measure of the Big Five factors (Costa and McCrae, 1992; Wiggins, 1996). Participants completed the 20-item Mini-IPIP using a 5-point scale (1 = *very inaccurate* and 5 = *very accurate*)—four items belonging to each of the following subscales: extroversion (e.g., “*Talk to a lot of different people at parties*”; *M* = 14.47, *SD* = 3.65, α = 0.81), agreeableness (e.g., “*Feel others' emotions*”; *M* = 16.32, *SD* = 2.95, α = 0.76), conscientiousness (e.g., “*Like order*”; *M* = 14.90, *SD* = 3.22, α = 0.70), neuroticism (e.g., “*Get upset easily*”; *M* = 11.81, *SD* = 3.54, α = 0.73), and intellect/imagination (e.g., “*Have vivid imagination*”; *M* = 14.26, *SD* = 3.14, α = 0.69), the latter being similar to the construct openness.

#### Narcissism

The Narcissistic Personality Inventory-16 [NPI-16; Ames et al., 2006 (NPI; Raskin and Terry, 1988)] is a psychometrically valid measure of subclinical narcissism (e.g., Konrath et al., 2014). Participants completed the NPI-16 using a 5-point Likert scale (1 = *strongly disagree* and 5 = *strongly agree*; 16 items [e.g., “*I am apt to show off if I get the chance*”]: *M* = 44.12, *SD* = 10.11, α = 0.89). The higher the score, the more narcissistic the individual is. The total score has been significantly correlated with expert ratings of narcissistic personality disorder (Miller and Campbell, 2008).

#### Self-esteem

The Rosenberg Self-Esteem Scale (RSES; Rosenberg, 1965) is a psychometrically valid instrument for the assessment of self-esteem (e.g., Huang and Dong, 2012). Participants completed the RSES using a 4-point Likert scale (0 = *strongly agree* and 3 = *strongly disagree*; 10 items [e.g., “*All in all, I am inclined to feel that I am a failure*”, “*I am able to do things as well as most other people*”]: *M* = 29.23, *SD* = 5.34, α = 0.89). The RSES assesses self-esteem as a single construct, and is designed to represent a global measure of perceived self-esteem of the participant's self-esteem. It measures both positive and negative feelings about the self. The five positive statements were recoded, meaning that a high composite score reflected high self-esteem.

### Data analysis

The dimensionality of the BYSAS was tested through a combination of exploratory (EFA) and confirmatory item factor analysis (CFA), conducted separately on the random split of the full sample. The objective of the exploratory analysis was to test the overall structure of the included items, with a particular focus on detecting deviations from the expected unidimensional structure. The objective of the CFA was to assess the goodness of fit of the unidimensional measurement model for the BYSAS. In the EFA, factor extraction criteria were very simple structure (VSS) (Revelle and Rocklin, 1979), and Velicer's (1976) minimum average partial (MAP) statistic. A bifactor rotation (Jennrich and Bentler, 2011) was used. The bifactor rotation enables the separation of a common factor and one or more specific factors. As noted by Reise et al. (2007), the bifactor model is particularly useful as a method to detect violations of undimensionality. In the context of testing unidimensional measurement models, the presence of specific factors in a bifactor model is a sign of local dependency within the factor. Such specific factors might be of substantive interest, but represents a violation of unidimensionality.

The results from the EFA-sample were fed into the CFA test of unidimensional model on the second split of the sample. The main objective of the CFA was to examine the fit of an unidimensional measurement model for the BYSAS, as well to test the discrimination and information from the set of items included. Global model fit was assessed through the Mplus robust weighted least square estimator. The root mean square error of approximation (RMSEA), the comparative fit index (CFI) and the Tucker-Lewis Index (TLI) were used as indicators of global model fit. For a good fit, these values should be < 0.06, > 0.95, and > 0.95, respectively (Hu and Bentler, 1999). We compared two classes of unidimensional item response theory (IRT) models: The Rasch partial credit model (Masters, 1982), and the graded response model (Samejima, 1997). To assess item fit to the Rasch partial credit model we assessed infit and outfit mean squares (Wright and Masters, 1982). According to conventional standards for survey research, infit, and outfit mean squares (MSQ) should preferably be in the range 0.6 to 1.4 (Wright and Linacre, 1994), but even numbers in the range 0.5 to 1.5 can be seen as “productive for measurement” (Linacre, 2002). A value below 1 means that the item responses are too predictable (overfit), whereas a value above 1 means the data responses are too random (underfit). The infit MSQ is weighted so that information close to the targeted item or person receive more weight.

To test invariance, differential item functioning (DIF) across gender and age groups was examined using a constrained stepdown approach, as implemented in the R mirt package (Chalmers, 2012). In the DIF analysis items were initially constrained to have equal discrimination and thresholds across groups. Statistically significant constraints were then released sequentially, using the remaining items as anchor items. This sequential stepdown procedure was first used on gender, treating males as the focal group, and females as the reference group. The same procedure was repeated for age groups, treating early adults (16–39 years) as the reference group and middle/late adulthood (40–88 years) as the focal group. The age group division was made as a compromise between age range (24 vs. 49 years) and number of participants in the groups (61.8% vs. 38.2%). Finally, the impact of DIF for test scores was assessed through differential test functioning (DTF) as defined by Meade (2010), and implemented by Chalmers et al. (2015).

The other analyses were conducted with SPSS, version 22. The BYSAS was evaluated in terms of internal consistency (Cronbach's alpha coefficient) and corrected item-total correlations, after transforming the variables into ranks in order to avoid the results being influenced by skewness (Greer et al., 2006). Correlation coefficients were calculated in order to assess the interrelationships between all study variables; *r* above 0.1, 0.3, and 0.5 were interpreted as small, medium and large effect size, respectively (Cohen, 1988). Differences in mean scores of BYSAS items between men and women were calculated; Cohen's *d* values of 0.2, 0.5, and 0.8 were defined as small, medium and large effects, respectively (Cohen, 1988).

In investigating factors related to sex addiction, a multinomial regression analysis was conducted based on the “no sex addiction” (score of zero) category (33.8% of the sample) as a reference. “Low sex addiction risk” (score of 1–6) comprised the second category (46.3% of the sample), “moderate sex addiction risk” (score of 7 or above) comprised the third category (19.1% of the sample), and “sex addiction” (score of 3 or 4 on at least four of the six BYSAS criteria) comprised the fourth category (0.7% of the sample). Independent variables consisted of gender, age, relationship status, education level, the five personality subscales of the Mini-IPIP, and the score on the NPI-16 and the RSES. Education was dummy coded so that the largest category (i.e., Bachelor's degree) comprised the reference category. In the analysis, each independent variable was included simultaneously. When the 95% confidence interval (CI) does not include 1.00, the result is regarded as statistically significant.

## Results

### Scale construction and development

Table 1 shows descriptive statistics of responses on the six BYSAS items. The mean score in the sample was 3.54 out of 24 (*SD* = 4.14). Items 1 (BYSAS_1_: salience/craving) and 2 (BYSAS_2_: tolerance) were more frequently endorsed in the higher rating category than other items. Men scored higher than women on all six BYSAS items, and the effect size (Cohen's *d*) of the difference in item mean scores between genders were 0.84 for salience/craving (large), 0.75 for tolerance (large), 0.41 for mood modification (medium–small), 0.69 for relapse/loss of control (medium–large), 0.65 for withdrawal (medium–large), and 0.36 for conflict/problems (medium–small).

The EFA suggested extraction of one factor according to the VSS criterion, but two factors according to Velicer's MAP criterion. The bifactor rotation of the two-factor solution revealed a strong general factor across all six items with loadings in the range 0.70 (BYSAS_1_) to 0.86 (BYSAS_4_ and BYSAS_6_) and an additional specific factor from BYSAS_1_ and BYSAS_2_. The specific factor could be interpreted as a local dependency between BYSAS_1_ and BYSAS_2_, and representing a violation of unidimensionality.

**Figure 1:**
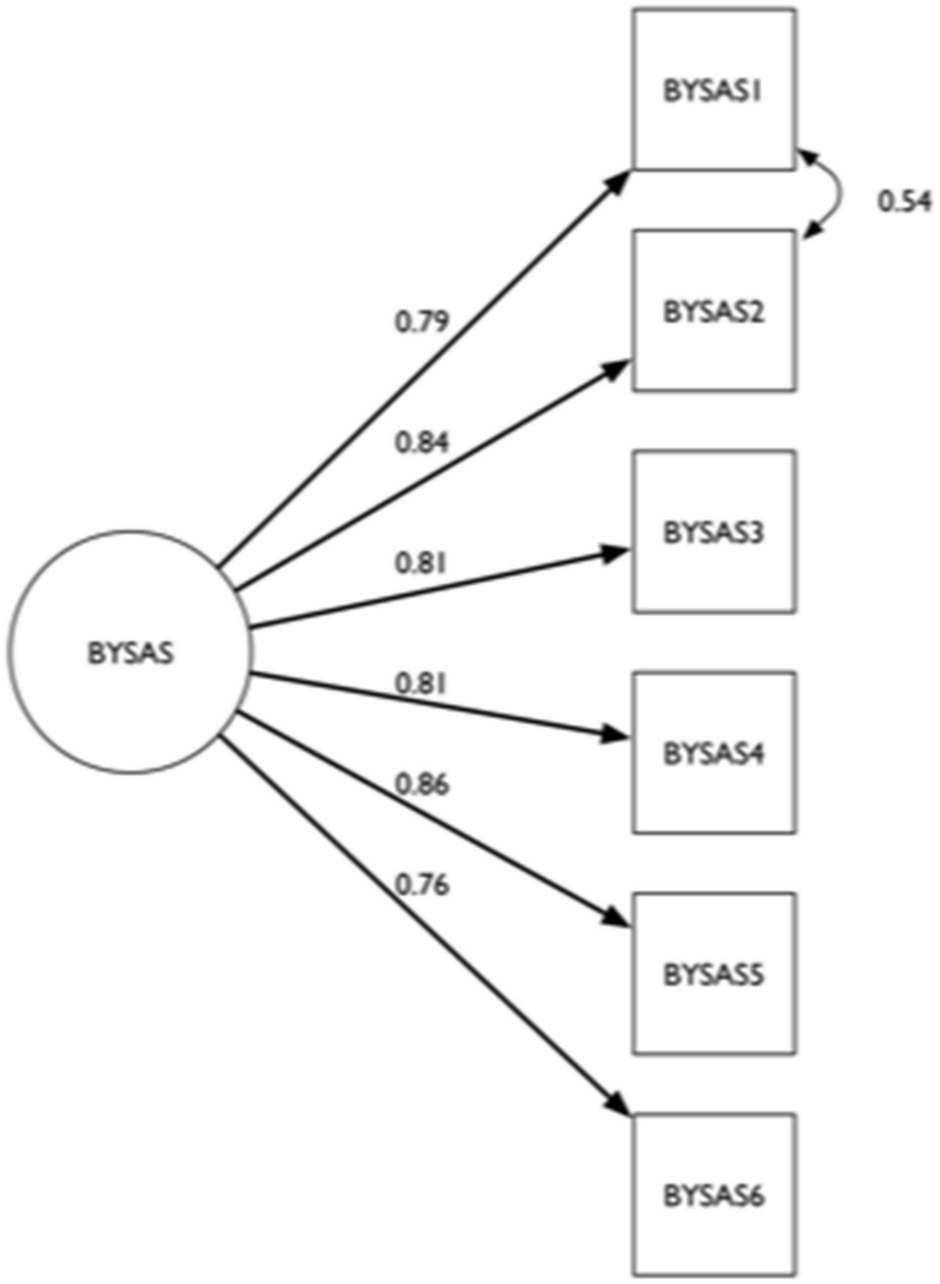
The factor structure of Bergen–Yale Sex Addiction Scale (BYSAS) showing standardized factor loadings for the CFA subsample (*n* = 11,766).

In line with the findings from the EFA, a one-factor model with correlated error terms for BYSAS^1^ and BYSAS^2^ was tested in a CFA with the Mplus robust weighted least square estimator for categorical data. The limited information fit statistics from the Mplus robust weighted least square estimation indicated an RMSEA of 0.046 [90% CI = 0.041, 0.051], a CFI of 0.998, and a TLI of 0.996, indicating high goodness of fit between the one-factor model and the data. Figure 1 shows the factor loadings based on the confirmatory subsample (*n* = 11,766).

To take into account the overlap between BYSAS_1_ and BYSAS_2_ in the unidimensional IRT models, a testlet of the sum of BYSAS_1_ and BYSAS_2_ was constructed. As the current items were highly skewed, the theta estimates were based on the empirical histogram method (Woods, 2007). Table 2 shows the infit and outfit mean squares (MSQ) from the partial credit model. All of the infit mean squares were in the desired 0.6 to 1.4 range (Wright and Linacre, 1994; Bond and Fox, 2015). The observed outfit MSQ for three items were lower than the prescribed 0.6 to 1.4 range in survey research, but were still in the range deemed “productive for measurement” (Linacre, 2002). The testlet outfit MSQ was 0.46. The borderline outfit MSQ values might reflect some degree of content redundancy in the testlet. That is, at a given score level, there is high consistency across item pairs, and too few “unexpected” responses. The infit MSQ values were in general closer to the expected value of 1, and could reflect that, although the responses were highly consistent, they were not deterministic in the Guttman sense of a strictly ordered sequence of item responses across the trait. The observed range of infit and outfit values indicated that the items of the BYSAS were reasonably in line with those predicted by the Rasch partial credit model. Still, model fit was better with the relaxed assumptions of the graded response model, as compared to the Rasch partial credit model (Akaikes information criterion PCM = 95155; Akaikes information criterion graded response model = 94843).

**Table 2:** Item fit statistics from Rasch partial credit model.

**Item**	**Infit MSQ**	**z.infit**	**Outfit MSQ**	**z.outfit**
BYSAS3	0.937	−3.430	0.696	−6.951
BYSAS4	0.942	−2.326	0.556	−7.082
BYSAS5	0.809	−10.684	0.575	−10.284
BYSAS6	0.916	−2.063	0.502	−6.545
Testlet BYSAS1 and 2	0.647	−26.029	0.459	−34.167

*BYSAS, Bergen-Yale Sex Addiction Scale; MSQ, mean square*.

Table 3 shows the results of tests of differential item functioning (DIF), and the estimated impact of DIF on item scores and expected total scores (differential test functioning; DTF). The first column shows change in chi-square when releasing assumptions of invariant slopes and intercepts. The sequential stepdown test of differential item functioning by gender indicated that BYSAS_3_ and BYSAS_4_ worked differently for males and females, with a significant drop in chi-square when releasing invariance constraints [BYSAS_3_: Chi-square (5) = 314.08, *p* < 0.001; BYSAS_4_: Chi-square (5) = 228.36, *p* < 0.001]. The DIF by age group identified BYSAS_3_ and BYSAS_4_ as items working differently by age groups [BYSAS_3_: Chi-square (5) = 67.28; BYSAS_4_: Chi-square (5) = 54.33]. For the other items, model constraints were not significant, indicating that the invariance assumption for these items was consistent with the data. Thus, the BYSAS satisfied the assumptions of *partial scalar equivalence* across gender and age groups.

**Table 3:** Test of differential item functioning and differential test functioning.

	**LRT DIF**	**df**	***p***	**SIDS/STDS**	**ESSD/ETSSD**
**GENDER (FEMALES REF.)**					
BYSAS3	314.083	5	<0.001	−0.281	−0.360
BYSAS4	228.358	5	<0.001	0.193	0.335
Impact total score				−0.088	−0.022
**AGE GROUP (YOUNG ADULTS REF.)**					
BYSAS3	67.289	5	<0.001	0.022	0.04
BYSAS4	54.334	5	<0.001	−0.018	−0.05
Impact total score				0.004	0.001

*LRT, likelihood-ratio test; DIF, differential item functioning; SIDS, signed item difference in the sample; STDS, signed test difference in the sample; ESSD, expected score standardized difference; ETSSD, expected test score standardized difference*.

**Figure 2:**
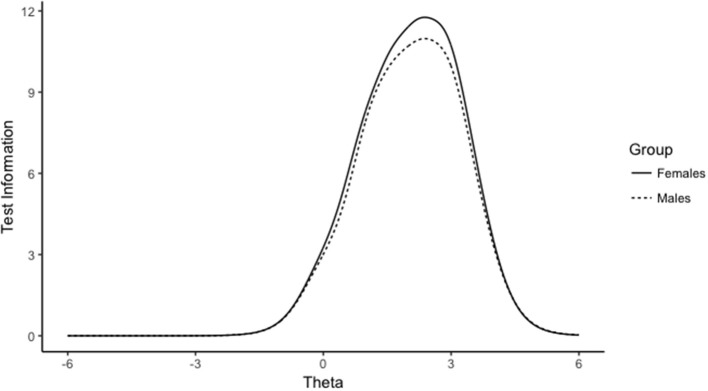
Test information curves from graded response model estimation of Bergen-Yale Sex Addiction Scale (*n* = 11,766).

The third and fourth column of Table 3 shows the effect size of DIF and DTF for BYSAS_3_ and BYSAS_4_, summarized through the signed item difference in the sample (SIDS/STDS) and the expected score standardized difference (ESSD/ETSSD). At the same level of trait, the average standard unit difference between males and females was −0.36 for BYSAS_3_ and 0.335 for BYSAS_4_. At the test level, these opposite effects canceled each other out, with a negligible differential test functioning for the expected total summed score. Similarly, for DIF by age group, the effect of BYSAS_3_ and BYSAS_4_ were in the opposite direction, canceling out the total effect. Young adults scored 0.04 standard units higher on BYSAS_3_, and 0.05 standard units lower on BYSAS_4_ compared to the middle/late adulthood group. At the test-level, the impact of DIF was only 0.0001 standard units, suggesting that the observed DIF for BYSAS_3_ and BYSAS_4_ did not have any impact on the total score level. To summarize, although DIF was observed for two items, the impact at the test level (DTF) was very small or ignorable. The test information curves for males and females are shown in Figure 2. The figure shows that the BYSAS had most information at very high levels of sex addiction (theta) for males and females, but very little information at lower levels of sex addiction.

### Reliability and internal consistency of the BYSAS

The Cronbach's alpha for the BYSAS was 0.83, and the corrected item-total correlation coefficients for Items 1 to 6 were 0.69 (BYSAS_1_: salience/craving), 0.74 (BYSAS_2_: tolerance), 0.62 (BYSAS_3_: mood modification), 0.57 (BYSAS_4_: relapse/loss of control), 0.66 (BYSAS_5_: withdrawal symptoms), and 0.42 (BYSAS_6_: conflict/problems), respectively.

### Convergent and discriminative validity

The correlation coefficient between the BYSAS's composite score and the sex subscale of the SPQ was 0.52. Table 4 shows that both of the scales demonstrated similar correlational patterns with other variables examined in the study. The zero-order correlation coefficients between study variables ranged from −0.53 (between self-esteem and neuroticism) to 0.52 (between the BYSAS and the SPQ-S).

**Table 4:** Zero-order correlation coefficients (Pearson product-moment correlation, point-biserial correlation, phi-coefficient) between variables.

	**Variables**	**1**	**2**	**3**	**4**	**5**	**6**	**7**	**8**	**9**	**10**	**11**	**12**	**13**	**14**	**15**	**16**	**17**
1	BYSAS	–																
2	SPQ–S	0.519																
3	Gender _(1 = ♂, 2 = ♀)_	−0.377	−0.252															
4	Age	−0.190	−0.086	0.031														
5	Relationship^a^	0.090	0.078	−0.065	−0.218													
6	Primary school	0.046	0.014	−0.028	−0.205	0.149												
7	High school	0.036	0.027	0.015	−0.197	0.094	−0.194											
8	Vocational school	0.028	0.028	−0.123	0.138	−0.049	−0.150	−0.263										
9	Bachelor's degree	−0.051	−0.032	0.095	0.118	−0.081	−0.231	−0.403	−0.313									
10	Master's degree	−0.040	−0.029	0.015	0.097	−0.073	−0.136	−0.237	−0.184	−0.282								
11	PhD degree	−0.014	−0.010	−0.018	0.057	−0.035	−0.036	−0.063	−0.049	−0.075	−0.044							
12	Extroversion	0.014	0.091	0.088	0.013	−0.064	−0.050	−0.019	−0.021	0.049	0.024	−0.001						
13	Agreeableness	−0.151	−0.147	0.343	0.048	−0.048	−0.049	−0.017	−0.060	0.073	0.031	0.001	0.296					
14	Conscientiousness	−0.208	−0.155	0.143	0.200	−0.130	−0.085	−0.052	0.052	0.033	0.041	−0.010	0.093	0.131				
15	Neuroticism	0.086	0.025	0.234	−0.116	−0.005	0.059	0.041	−0.021	−0.024	−0.041	−0.022	−0.098	0.093	−0.157			
16	Intellect/imagination	0.093	0.075	−0.105	−0.036	0.043	−0.045	−0.042	−0.066	0.026	0.109	0.062	0.163	0.116	−0.116	−0.003		
17	Narcissism	0.213	0.213	−0.219	−0.125	−0.003	−0.023	−0.039	−0.049	0.034	0.067	0.009	0.370	−0.075	0.026	−0.150	0.196	
18	Self-esteem	−0.092	−0.016	−0.140	0.154	−0.125	−0.124	−0.104	0.017	0.072	0.109	0.037	0.315	0.055	0.296	−0.530	0.113	0.416

*N = 23,533. BYSAS, Bergen–Yale Sex Addiction Scale; SPQ-S, Shorter PROMIS Questionnaire—Sex scale*.

a *1 = in a relationship, 2 = not in a relationship*.

*−0.012 ≤ r ≤ 0.012—ns, −0.016 ≤ r ≤ −0.013 or 0.13 ≤ r ≤ 0.016—p < 0.05, −0.017 ≥ r or r ≥ 0.017—p < 0.01*.

### Relations with demographics, big five, narcissism, and self-esteem

The independent variables explained 23.0% (Cox–Snell formula) of the variance in sex addiction risk (26.0% according to Nagelkerke formula; see Table 5). The odds of belonging to the “low sex addiction risk”, the “moderate sex addiction risk” and the “sex addiction“ categories were higher for men than for women. Age was inversely related to sex addiction category. Not being in a relationship increased the odds of belonging to the “moderate sex addiction risk” category. Primary school education lowered the odds of belonging to the “low sex addiction risk” and the “moderate sex addiction risk” categories. Having a Master's degree lowered the odds of belonging to the “moderate sex addiction risk” category while having a PhD degree increased the odds of belonging to the “sex addiction” category. Extroversion increased the odds of belonging to the three upper sex addiction categories, whereas conscientiousness lowered the corresponding odds. Agreeableness lowered the odds of belonging to the “sex addiction” category. Neuroticism increased the odds of belonging to the “moderate sex addiction risk” and the “sex addiction” categories. Intellect/imagination was positively associated with belonging to the “low sex addiction risk” and the “moderate sex addiction risk” categories. Self-esteem was inversely related to the sex addiction categories. Finally, narcissism was positively associated with belonging to the three upper sex addiction categories.

**Table 5:** Multinomial logistic regression of sex addiction (reference category: BYSAS score of 0; *OR* = 1.00; *n* = 7,962).

	**Low sex addiction risk** **(BYSAS score 1–6; *n* = 10,907)**	**Moderate sex addiction risk** **(≥ 7/< 4 criteria fulfilled; *n* = 4,490)**	**High sex addiction risk—sex addiction** **(Fulfilling 4–6 criteria; *n* = 174)**
**Independent variable**	***OR*** **(95% CI)**	***OR*** **(95% CI)**	***OR*** **(95% CI)**
Gender _(1 = ♂, 2 = ♀)_	**0.272 (0.250–0.295)**	**0.081 (0.073–0.090)**	**0.035 (0.023–0.051)**
Age	**0.982 (0.980–0.985)**	**0.968 (0.965–0.972)**	**0.956 (0.941–0.972)**
Relationship _(1 = *in*, 2 = *not in*)_	1.045 (0.977–1.118)	**1.105 (1.010–1.210)**	1.030 (0.738–1.437)
Education _(reference=Bachelor's degree)_			
Primary School	**0.752 (0.669–0.845)**	**0.694 (0.595–0.809)**	1.238 (0.740–2.071)
High School	0.984 (0.906–1.069)	0.964 (0.860–1.080)	1.083 (0.680–1.727)
Vocational School	1.034 (0.942–1.136)	1.066 (0.940–1.210)	1.299 (0.782–2.158)
Master's degree	0.953 (0.867–1.047)	**0.848 (0.740–0.971)**	1.022 (0.554–1.884)
PhD degree	0.777 (0.587–1.030)	0.737 (0.493–1.102)	**3.229 (1.071–9.734)**
Extroversion	**1.030 (1.020–1.040)**	**1.045 (1.031–1.059)**	**1.059 (1.010–1.111)**
Agreeableness	1.008 (0.995–1.020)	0.988 (0.973–1.004)	**0.946 (0.900–0.995)**
Conscientiousness	**0.958 (0.948–0.969)**	**0.915 (0.903–0.928)**	**0.886 (0.844–0.930)**
Neuroticism	1.010 (0.999–1.021)	**1.097 (1.081–1.113)**	**1.249 (1.183–1.319)**
Intellect/imagination	**1.015 (1.004–1.025)**	**1.025 (1.010–1.039)**	1.002 (0.951–1.055)
Self-esteem	**0.976 (0.968–0.984)**	**0.928 (0.918–0.939)**	**0.858 (0.829–0.888)**
Narcissism	**1.027 (1.023–1.030)**	**1.059 (1.054–1.065)**	**1.091 (1.072–1.111)**

*Significant findings in bold. OR, odds ratio; CI, confidence interval; BYSAS, Bergen–Yale Sex Addiction Scale*.

## Discussion

Although problematic sexual behavior has been argued as representing an addictive disorder, previously developed screening tools assessing the disorder have not included core addiction criteria. Consequently, the BYSAS was developed in order to overcome this limitation and its psychometric properties were examined in a large national sample. To ensure content validity, the construction process was based on components that theoretically reflect all core dimensions of addiction. Rigorous analyses demonstrated that the BYSAS has good psychometrics, and are discussed further below.

A one-factor model with an added specific correlation between salience (BYSAS_1_) and tolerance (BYSAS_2_) error terms achieved a high goodness of fit to the observed data. According to this model an increase in sex addiction increases the probability of endorsing each of the key characteristics of addiction, and the high factor loading indicated that each indicator was tapping information about the underlying addiction. While suggesting one dominant factor, the local dependence between salience and tolerance warrants some attention. Considering the content of these two items, the residual correlation is not primarily about logical consistency, but might reflect a specific motivational overlap, in that salience might contribute to increased sex urge. In the context of practical scale administration, the local dependence is less of importance, as the sum of items essentially reflects one dimension. The high goodness of fit for the one-factor model and the uniformly high factor loadings suggested that the BYSAS reflects one single construct. Consequently, Hypothesis 1 and 2 were supported by results of the data analysis. In terms of the DIF analyses males had scored higher than females on BYSAS_4_ and lower on BYSAS_3_ whereas young adults (16–39 years) scored higher on BYSAS_3_ and lower on BYSAS_4_ compared to older adults (40 to 88 years). At the test level these effects overall canceled each other, thus the impact at the test level was ignorable.

There was a significant and positive correlation (0.52) between scores on the BYSAS and the SPQ-S (Christo et al., 2003). This high correlation indicates the BYSAS's convergent validity and provides support for Hypothesis 3. Results also demonstrated that the BYSAS and the SPQ-S showed similar correlations with other variables examined in the present study. However, further studies examining the convergent validity and test-retest reliability of the BYSAS are needed. The distribution of the scores of the BYSAS was strongly skewed to the left (i.e., low scores), which is as expected because the BYSAS assessed sex addiction symptoms in a large unselected population-based sample. Salience/craving and tolerance were more frequently endorsed in the higher rating category than other items, and these items had the highest factor loadings. This seems reasonable as these reflect less severe symptoms (e.g., question about depression: people score higher on feeling depressed, then they plan committing suicide). This may also reflect a distinction between engagement and addiction (often seen in the game addiction field)—where items tapping information about salience, craving, tolerance, and mood modification are argued to reflect engagement, whereas items tapping withdrawal, relapse and conflict more measure addiction. Another explanation could be that salience, craving, and tolerance may be more relevant and prominent in behavioral addictions than withdrawal and relapse.

In terms of demographics, results from the multivariate analyses concur with findings from previous studies (Kafka, 2010; Karila et al., 2014; Campbell and Stein, 2015; Wéry et al., 2016a; Wéry and Billieux, 2017), and supported Hypothesis 4. A high score on the BYSAS was associated with being male and men scored higher than women on all six BYSAS items, which suggest that men are more at risk than women in developing sex addiction. This also corresponds to the fact that the majority of individuals seeking professional help for addictive sex behavior are men (Kafka, 2010; Griffiths and Dhuffar, 2014; Campbell and Stein, 2015). To some extent, this might also reflect that women to a lesser degree come forward due to potentially more social stigma and inner shame than men (Gilliland et al., 2011; Dhuffar and Griffiths, 2014, 2015). Age was inversely related with sex addiction, and corresponds to empirical evidence showing that being of a young age is a vulnerability factor for developing and maintaining addictions in general (Chambers et al., 2003). Additionally, given that some types of excessive sex can be physically demanding and that sexual libido tends to decrease as individuals get older, it is perhaps unsurprising that sex addiction is associated with younger age.

Not being in a relationship was also associated with sex addiction, possibly because single individuals are more motivated to satisfy unmet sexual needs than those in a stable relationship (Ballester-Arnal et al., 2014; Sun et al., 2014). Another explanation may be that “sex addicts” have difficulties in establishing and maintaining relationships (e.g., childhood trauma, insecure attachment, etc.; Dhuffar and Griffiths, 2015; Weinstein et al., 2015). The present results also showed that, compared to the reference category (having a Bachelor's degree), those with higher education (i.e., having a PhD) were more likely to have a high BYSAS score. Given that education is related to high social status, it may be that such individuals gain access to more sexual opportunities, especially in men (Buss, 1998). However, we explored the interaction effects (Gender x PhD), none of which turned out significant (Gender x Bachelor as contrast; results not shown). Still, future studies should examine Gender x Education interactions regarding sex addiction.

Scores on the BYSAS had positive associations with neuroticism, extroversion, and intellect/imagination, and negative associations with agreeableness and conscientiousness. Overall, the results from the multivariate analyses were as expected, and support the discriminant validity of BYSAS (Hypothesis 5). The positive relationship with extroversion may reflect extroverts' tendency to seek stimulation in the company of others, and their concern about individual expression and the enhancement of personal attractiveness (Costa and Widiger, 2002). Their social nature may also increase the potential of more sexual opportunities (e.g., socializing at parties, leisure events, etc.). The positive relationship with neuroticism also corroborates findings from previous studies (Pinto et al., 2013; Rettenberger et al., 2016; Walton et al., 2017), and is congruent with the assumption that sex has an anxiolytic effect (Coleman, 1992), and that engaging in sexual activities may function as an escape from dysphoric feelings (O'Brien and DeLongis, 1996; Dhuffar et al., 2015; Wéry et al., 2016b). Intellect/imagination also had a positive relationship with addictive sexual behavior. This may reflect the fact that people scoring high on this trait tend to pursue self-actualization by seeking out intense, unusual, and/or euphoric experiences, such as specific sex behaviors—and their holding of a liberal belief system (Costa and Widiger, 2002). Conscientiousness and agreeableness were inversely related to sex addiction, which may be explained by the fact that these traits reflect features such as self-control and the ability to resist temptations, and putting other interests before one's own, and being sensitive and good-natured. Taken together, the current findings support the notion that agreeableness and conscientiousness (in general) protects against addictions, whereas extroversion and neuroticism (Few et al., 2014) facilitate them—findings that have been reported elsewhere (e.g., Hill et al., 2000; Kotov et al., 2010; Maclaren et al., 2011; Andreassen et al., 2013; Walton et al., 2017).

The present study also found sex addiction to be positively associated with narcissism, and negatively associated with self-esteem, supporting both Hypothesis 6 and previous studies (Kafka, 2010; Kor et al., 2014; Kasper et al., 2015; Doornwaard et al., 2016). These findings indicate that sexual behavior may be a way of counteracting low self-esteem and enhancing higher self-esteem (e.g., associated effects from being sexually active including feelings of being popular, receiving compliments, feelings of omnipotence when engaging in sex, being given attention during sex, etc.), escaping from low self-esteem feelings, or that addictive sex reduces self-esteem. Narcissistic tendencies and sex addiction have consistently co-varied in previous studies (Black et al., 1997; Raymond et al., 2003; Kafka, 2010; MacLaren and Best, 2013; Kasper et al., 2015), and might reflect that sex behavior is a manifestation of narcissistic traits (e.g., desire for attention, admiration, and power, exploitation and sense of entitlement, etc.). Another possibility is that excessive sexual behavior fosters narcissistic traits among those that have high numbers of sexual partners.

### Limitations and strengths of the present study

The present study is limited by all the common shortcomings of self-report data and self-selecting sampling methodology (e.g., self-selection bias, unknown response rate, and lack of information about non-respondents). As the scores on the BYSAS had a right skewed distribution, a risk of floor effects influencing the results (e.g., lowering relationships between constructs) was present. However, the full range of scores on all variables was presented in the data, which strengthens the validity of estimated relationship between the constructs investigated. It should also be noted that about one-quarter of the variance in the multinomial regression analysis was explained by the independent variables. The creation of four categories of levels of sex addiction made in the present study should be regarded as tentative because no well-defined cut-offs or agreed upon diagnostic criteria exist. This also prevented us from using receiver operating characteristics curve analysis where cut-offs can be evaluated in terms of sensitivity and specificity against a “gold standard.” The cross-sectional study design may have influenced the results due to factors such as the common method bias, thus creating inflated relationships between the variables examined in the present study (Podsakoff et al., 2003). Furthermore, due to the large sample size providing power to the analyses, several small correlations may have turned out significant. Although some of the significant findings may reflect trivial relationships due to the large sample size, some effect sizes in the correlation analysis were moderate to large suggesting some substantial and meaningful relationships between study variables (Cohen, 1988).

Although the survey completion was anonymous, reporting problematic sexual behaviors may be associated with shame and taboo (Dhuffar and Griffiths, 2014), and might have caused socially desirable answers. Also, voluntarily responding to an online newspaper article about excessive behaviors may possibly have attracted specific types of individual (e.g., those that used the Internet excessively, younger individuals). However, attracting such individuals may arguably have also been an advantage because having individuals in the sample that have addictive problems may have strengthened the scale's validity for use in clinical contexts. Further studies psychometrically testing the BYSAS's properties are needed, especially in terms of test-retest reliability and its cultural adaptability and generalizability.

The selection of measures may have also limited the present study, because other psychometrically valid scales that assess problematic sex were not used in comparison to the BYSAS. For example, the Hypersexual Disorder Questionnaire (HDQ; Reid et al., 2012) is a comprehensive assessment measure including the proposed diagnostic criteria for hypersexual disorder (Kafka, 2010). However, the proposed *DSM-5* criteria do not fully reflect core addiction elements such as tolerance, withdrawal, and mood modification. Thus, it was deemed more appropriate to compare the BYSAS to a scale developed using addiction theory and criteria.

The extremely large sample size in the present study is one of the key strengths in providing high statistical power in relation to all the analyses conducted. The findings complement many of the previous small-scale and population-specific studies in the field. Another strength of the present study is the inclusion of specific and core addiction criteria in the scale construction and development process and the use of relevant constructs and validated instruments in the validation process. Also, the BYSAS takes into account the concept of craving (wanting/craving state), which is now added in the *DSM-5* (American Psychiatric Association, 2013) as an addiction symptom. Additionally, the BYSAS is more of a generic sex addiction screening instrument, because it does not focus on particular demographic groups (e.g., male, gay) or medium (e.g., online sex). Consequently, the BYSAS can be used to assess both online and offline sexual activity and is arguably more suited to assessing contemporary sexual behavior. Another key strength was that the study was advertised nationally rather than locally (in the national press). The national press in Norway is known for having a wide demographic audience compared to local press. Therefore, the sample is probably more representative of the Norwegian population and is arguably more representative than other studies using self-selected samples. This is also one of the few studies in this field that focuses on the general population, and comprises a great proportion of women as well. Furthermore, the brevity of this new scale makes it suitable to be included in space-limited surveys.

## Conclusions

In the present study a new scale for assessing addictive sex behavior, the BYSAS, was developed. Reliability and of the BYSAS were established with a national sample of 23,533 Norwegian adults. The assumed one-factor structure was confirmed by EFA and CFA, and the internal consistency was high. By including items covering all core addiction symptoms, content validity was ensured. The BYSAS was validated against another sex addiction measure, as well as measures of demographics, personality, and self-esteem; and a tentative cut-off score is proposed. Overall, the BYSAS is a psychometrically sound and valid instrument to measure sex addiction, which may be used freely by researchers and practitioners in epidemiological studies and treatment settings.

## Author contributions

CA: Contributed to the conception and design of the work, the acquisition, analysis, and interpretation of data; TT: Contributed to the analysis; SP, MG, TT, and RS: Contributed to the interpretation of data for the work; CA: Drafted the work; All authors revised the work critically in terms of important intellectual content; All authors approved the final version and are accountable for all aspects of the work in terms of ensuring that questions related to the accuracy or integrity of any part of the work were appropriately investigated and resolved.

### Conflict of interest statement

The authors declare that the research was conducted in the absence of any commercial or financial relationships that could be construed as a potential conflict of interest.

## Footnotes

^1^The set of criteria (Preoccupied, Ashamed, Treatment, Hurt others, Out of control, Sad) is based on the acronym PATHOS, which the Greeks used for “suffering”.

## Appendix A

### Bergen–yale sex addiction scale

**Table:** Below are some questions about your relationship to sex/masturbation. (NB! By sex means here different sexual fantasies, urges and behaviors such as masturbation, pornography, sexual activities with consenting adults, cybersex, telephone sex, strip clubs, and the like). Choose the response alternative for each question that best describes you.

	***How often during the past year have you**…*	**Very rarely**	**Rarely**	**Sometimes**	**Often**	**Very often**
1.	Spent a lot of time thinking about sex/masturbation or planned sex?	❑	❑	❑	❑	❑
2.	Felt an urge to masturbate/have sex more and more?	❑	❑	❑	❑	❑
3.	Used sex/masturbation in order to forget about/escape from personal problems?	❑	❑	❑	❑	❑
4.	Tried to cut down on sex/masturbation without success?	❑	❑	❑	❑	❑
5.	Become restless or troubled if you have been prohibited from sex/masturbation?	❑	❑	❑	❑	❑
6.	Had so much sex that it has had a negative impact on your private relationships, economy, health, and/or job/studies?	❑	❑	❑	❑	❑

All items are scored along the following scale: 0 = Very rarely, 1 = Rarely, 2 = Sometimes, 3 = Often, 4 = Very often

## Appendix B

### Shorter PROMIS questionnaire–sex subscale

**Table:** Below are some questions about your relationship to sex. Choose the response alternative for each question that best describes you^a^

***Answers should be given for life-time use rather than just recent use i.e., have you ever…***		**Not like me at all**					**Most like me**
		**0**	**1**	**2**	**3**	**4**	**5**
1.	I find it difficult to pass over an opportunity for casual or illicit sex	❑	❑	❑	❑	❑	❑
2.	Others have expressed repeated serious concern over my sexual behavior	❑	❑	❑	❑	❑	❑
3.	I pride myself on the speed with which I can get to have sex with someone and find that sex with a complete stranger is stimulating	❑	❑	❑	❑	❑	❑
4.	I would take an opportunity to have sex despite having just had it with somebody else	❑	❑	❑	❑	❑	❑
5.	I find making a sexual conquest causes me to lose interest in that partner and leads me to begin looking for another	❑	❑	❑	❑	❑	❑
6.	I tend to ensure that I have sex of one kind or another rather than wait for my regular partner to be available again after an illness or absence	❑	❑	❑	❑	❑	❑
7.	I have had repeated affairs even though I had a regular relationship	❑	❑	❑	❑	❑	❑
8.	I have had three or more regular sexual partners at the same time	❑	❑	❑	❑	❑	❑
9.	I have had voluntary sex with someone that I dislike	❑	❑	❑	❑	❑	❑
10.	I tend to change partners if sex becomes repetitive	❑	❑	❑	❑	❑	❑

Source: From How to Identify Addictive Behaviour by R. Lefever, 1988, London, UK: PROMIS Publishing. [This is the source reference for the PROMIS questionnaire, from which the items for the sex subscale were taken.]. Copyright by PROMIS Clinics. Reprinted with kind permission from R. Lefever (personal communication, March 14, 2017).

a *Instruction wording used in the current study, and not from the SPQ*.
